# Two new species of *Plectranthias* (Teleostei, Serranidae, Anthiadinae) from mesophotic coral ecosystems in the tropical Central Pacific

**DOI:** 10.3897/zookeys.941.50243

**Published:** 2020-06-16

**Authors:** Bart Shepherd, Tyler A. Y. Phelps, Hudson T. Pinheiro, Claudia R. Rocha, Luiz A. Rocha

**Affiliations:** 1 Steinhart Aquarium, California Academy of Sciences, San Francisco, CA 94118, USA; 2 Department of Ichthyology, California Academy of Sciences, San Francisco, CA 94118, USA; 3 Department of Biology, San Francisco State University, San Francisco, CA 94132, USA

**Keywords:** biodiversity, closed-circuit rebreather, coral-reef twilight zone, ichthyology, perchlet, reef fish, taxonomy

## Abstract

Two new species of *Plectranthias* perchlets are described, collected from mesophotic coral ecosystems in French Polynesia and the Republic of the Marshall Islands, in the tropical Central Pacific. *Plectranthias polygonius***sp. nov.** was collected at a depth of 105 m in Tahiti, French Polynesia, and 120 m in Maloelap Atoll, Republic of the Marshall Islands. It was also observed in Moorea and Rangiroa (French Polynesia), and at Majuro and Erikub Atolls, Republic of the Marshall Islands. *Plectranthias hinano***sp. nov.** was collected at a depth of 90–98 m in Tahiti, French Polynesia, and observed in Moorea. The barcode fragment of the cytochrome oxidase I gene of *Plectranthias polygonius***sp. nov.** does not closely match any published sequence of *Plectranthias*, with approximately 15% uncorrected divergence from several species. *Plectranthias polygonius***sp. nov.** can be distinguished from all of its congeners by coloration and morphology. The barcode fragment of the COI gene of *Plectranthias hinano***sp. nov.** is closest to *Plectranthias bennetti*, with 5.4% uncorrected divergence. *Plectranthias hinano***sp. nov.** is also distinguished from all of its congeners by morphology, and a coloration that includes two indistinct black spots along the base of the dorsal-fin, and transparent yellow dorsal and anal fin membranes. With this publication, the genus *Plectranthias* now comprises 58 valid species, with representatives from tropical to temperate waters of the Atlantic, Pacific, and Indian oceans. These two new discoveries add to the growing body of research highlighting the rich biodiversity of mesophotic ecosystems.

## Introduction

The anthiadine genus *Plectranthias* Bleeker, 1873, currently comprises 56 valid species from tropical to temperate waters in the Atlantic, Pacific, and Indian oceans (Fricke et al. 2019). Most of these fishes are found in relatively deep habitats (depths of 90–420 m) with complex rocky formations (Allen and Walsh 2015; Gill et al. 2016). In general, they are small (20 cm max length, but most in the 5–10 cm range), benthic, feed on small mobile invertebrates, and hide in crevices and holes on the reef (Kuiter 2004). Due to their small size and cryptic habits, they are poorly represented in museum collections, and most species have been described based on a single or a small number of specimens (Randall 1980; Heemstra and Randall 2008; Bineesh et al. 2014; Allen and Walsh 2015; Gill et al. 2016; Shepherd et al. 2018; Gill and Roberts 2020; Wada et al. 2020).

Mesophotic coral ecosystems (MCEs) are coral reef habitats found at depths of 30–150 m, and are also known as the coral reef “twilight zone” (Rocha et al. 2018). While conducting ichthyological and ecological surveys of MCEs at various locations across the wider tropical Pacific region, our team has encountered many new species, especially reef fishes within the family Serranidae. In this paper, we describe two new anthiadine fishes within the genus *Plectranthias* from French Polynesia and the Republic of the Marshall Islands. These two new perchlets represent the 57^th^ and 58^th^ species of *Plectranthias*, adding to the spate of recent new species from MCEs.

## Materials and methods

All specimens were collected with hand nets while diving on mixed-gas closed-circuit rebreather (Hollis Prism 2) in French Polynesia (Tahiti, Moorea) and Micronesia (Majuro, Republic of Marshall Islands). Specimens were collected and immediately transported to a field laboratory, where they were photographed, tissues sampled, fixed in 10% formalin, and preserved in 75% ethanol. The preserved specimens were later measured and x-radiographed at the California Academy of Sciences. Measurements were taken with digital calipers to the nearest 0.01 mm and rounded to one decimal place, following the conventions described in Anderson and Heemstra (2012), Williams et al. (2013), and Gill and Roberts (2020). Principal caudal rays are those associated with hypurals, as described in Gill et al. (2016). Procurrent caudal-fin rays are those dorsal and ventral to the principal rays. Principal and procurrent caudal-fin ray counts are presented as upper + lower. Vertebral counts are presented as precaudal + caudal. The anterior-most vertebra with a haemal spine was counted as the first caudal vertebra, the urostylar complex the last. Gill raker counts are presented as upper (epibranchial) + lower (ceratobranchial) rakers on the anterior face of the first arch; the angle raker is included in the second count. The anterior supraneural-dorsal ray-pterygiophore-neural spine interdigitation pattern is presented as a formula with “0” representing a supraneural, “/” a neural spine, and numerals indicating the number of spines borne by each pterygiophore (Anderson and Heemstra 2012; Williams et al. 2013). Morphometric and meristic data for the holotypes and paratypes are presented in Table 1. Measurements in the text are proportions of standard length ( xlink:title="standard length" SL), unless otherwise noted. Values in parentheses represent ranges for paratypes, when different from the holotypes. The holotypes were deposited at the California Academy of Sciences ichthyological collection (**CAS**), and the paratypes were deposited at the Smit hsonian Institution, National Museum of Natural History ichthyological collection (**USNM**).

**Table 1.** Proportional measurements of type specimens of *Plectranthias polygonius* sp. nov., and *Plectranthias hinano* sp. nov. expressed as a percentage of the standard length.

	*Plectranthias polygonius* sp. nov.		*Plectranthias hinano* sp. nov.	
	Holotype	Paratype	Holotype	Paratype
	CAS 247193	USNM 445722	CAS 247195	USNM 445723
Standard length (mm)	29.5	32.3	49.6	28.0
Head length	31.3	32.3	42.1	39.8
Greatest body depth	29.5	31.6	34.0	33.2
Body width	11.1	13.5	16.5	13.9
Snout length	9.3	9.0	14.2	12.3
Postorbital of head	21.0	21.1	18.6	17.9
Bony interorbital width	5.9	7.1	4.0	5.4
Orbit diameter	11.2	11.5	11.5	11.4
Upper jaw length	17.6	19.1	19.0	19.1
Maxilla width	5.6	6.2	5.9	5.6
Caudal peduncle length	8.7	8.1	10.6	9.7
Caudal peduncle depth	12.3	13.0	11.4	11.8
Predorsal length	38.0	41.4	41.8	41.0
Preanal length	70.0	70.6	68.4	67.4
Prepelvic length	35.9	38.7	42.5	39.9
Dorsal-fin base length	48.7	52.9	48.5	53.7
First dorsal spine	5.6	6.3	5.7	10.7
Longest dorsal spine (number)	18.8 (3rd)	20.0 (3rd)	18.2 (4th)	15.7 (3rd)
First segmented dorsal ray	17.1	15.6	14.9	16.4
Longest segmented dorsal ray (number)	17.3 (2nd)	15.6 (1st)	15.3 (2nd)	19.8 (3rd)
Anal fin base length	15.8	15.4	16.0	18.8
First anal spine	7.6	8.0	6.3	8.0
Second anal spine	19.0	19.6	20.6	20.8
Third anal spine	13.2	13.7	15.9	15.4
First segmented anal ray	17.5	17.7	16.1	17.7
Longest anal spine (number)	25.8 (2nd)	23.5 (2nd)	15.4 (2nd)	27.1 (2nd)
Longest segmented anal ray (number)	23.6 (4th)	21.5 (4th)	14.0 (2nd)	24.8 (2nd)
Caudal-fin length	20.9	21.7	32.5	27.9
Pectoral-fin length	26.2	29.7	35.7	38.7
Pelvic spine length	16.0	16.5	17.4	17.4
Pelvic-fin length	21.8	23.6	21.5	24.9

Mitochondrial cytochrome c oxidase subunit I (COI) DNA was sequenced and analyzed for the new species. DNA extraction and PCR amplification of the COI gene were performed following protocols detailed in Weigt et al. (2012). DNA sequences were compared to the fourteen *Plectranthias* species available in GenBank (*P. ahiahiata* Shepherd, Phelps, Pinheiro, Perez-Matus & Rocha, 2018: MH025944; *P. alleni* Randall, 1980: FOAO1479; *P. bennetti* Allen & Walsh, 2015: KT601636; *P. flammeus* Williams, Delrieu-Trottin & Planes, 2013: KC565477–KC565480; *P. fourmanoiri* Randall, 1980: KC567662, KC567663; *P. japonicus* Steindachner, 1883: JQ681323, JQ681324; *P. kamii* Randall, 1980: KU943548; *P. kelloggi* Jordan & Evermann, 1903: KP267643; *P. longimanus* Weber, 1913: JF494178; *P. maculicauda* Regan, 1914: FNZ095; *P. nanus* Randall, 1980: JQ432001–JQ432004, KC565481, KC567661; *P. randalli* Fourmanoir & Rivaton, 1980: KP267613; *P. retrofasciatus* Fourmanoir & Randall, 1979: JN313133; *P. winniensis* Tyler, 1966: KC565482, KC565483). Alignments of DNA sequences were done using a standard Geneious global alignment with free end gaps and 65% similarity in the program Geneious Prime 2020.0.3 (Biomatters, Auckland; Kearse et al. 2012).

## Taxonomy

### Plectranthias polygonius sp. nov.

Taxon classificationAnimaliaPerciformesSerranidae

201F672B-E681-5F93-94AC-0AC9F8959557

http://zoobank.org/12603BD5-EA0F-4826-AA9C-A380A594F316

**Figure 1.**
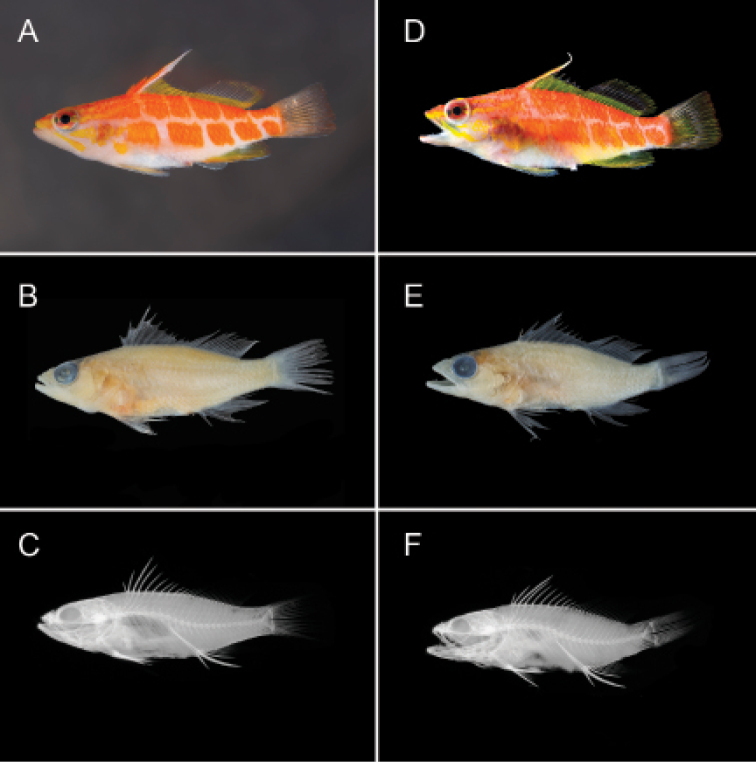
*Plectranthias polygonius* sp. nov. holotype **A** shortly after death **B** preserved **C** radiograph, and paratype **D** shortly after death **E** preserved **F** radiograph. Photographs by LA Rocha (**A**), A Gaisiner (**B, E**), J Fong (**C, F**), and T Sinclair-Taylor (**D**).

**Figure 2.**
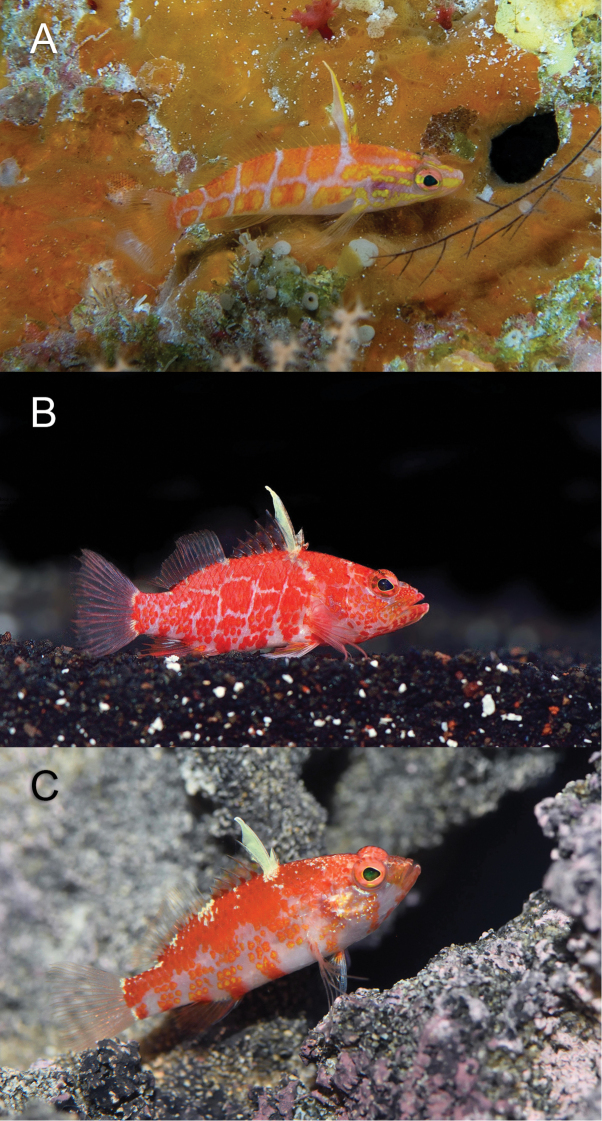
*Plectranthias polygonius* sp. nov. at Erikub Atoll, Republic of the Marshall Islands, at a depth of 120 m (**A**), aquarium photos of *P. inermis* (**B**), and *P. altipinnatus* (**C**). Photographs by LA Rocha (**A**) and YK Tea (**B, C**).

Figures 1 , 2A , Table 1 Polygon Perchlet

#### Type locality.

Tahiti, French Polynesia.

#### Holotype.

CAS 247193, field code: HTP906, GenBank MN922331. 29.5 mm SL, Tahiti, French Polynesia, 17°29'27"S, 149°28'01"W, depth of collection 105 m, collected with hand nets by B Shepherd, HT Pinheiro, TAY Phelps, MV Bell, and LA Rocha, 03 March 2019.

#### Paratype.

USNM 445722, field code: HTP942, GenBank MN922330. 32.3 mm SL, Maloelap Atoll, Republic of the Marshall Islands, 8°37'42"N, 170°59'58"E, depth of collection 120 m, collected with hand nets by HT Pinheiro, TAY Phelps, MV Bell, and LA Rocha, 13 August 2019.

#### Diagnosis.

*Plectranthias polygonius* sp. nov. can be distinguished from all of its congeners by live coloration, in particular the two rows of orange rhomboid-shaped polygons on the lateral part of the body and an elongated yellow and white third dorsal spine (Fig. 1A, D), and by the following combination of characters: dorsal-fin rays X, 16; pectoral-fin rays 14, all unbranched; vertebrae 10+16; continuous lateral line with 27–30 tubed scales; circumpeduncular scales 10 or 11; absence of antrorse spines on the preopercle.

#### Description.

Proportional measurements for the type specimens are presented in Table 1. Dorsal rays X, 16, the last soft ray branched to base and counted as one; first dorsal spine short, 18.0 in SL (15.8); third dorsal spine longest, 1.7 in HL (1.6) with flag-like extension; dorsal-fin base length 2.1 in SL (1.9); anal-fin rays III, 7, last soft ray branched to base and counted as one; anal-fin base length 6.3 in SL (6.5); second anal spine longest and stoutest at 1.6 in HL; anal-fin origin at vertical beneath fourth dorsal-fin ray; pectoral-fin rays 14, all unbranched, length 3.8 in SL (3.4); pelvic fin I, 5; pelvic-fin length 4.6 in SL (4.2); pelvic-spine length 2.0 in HL; procurrent caudal-fin rays 7+6 (6+5); principal caudal-fin rays 9+8.

Body moderately elongate, laterally compressed; depth of body 3.4 in SL (3.2); width of body 2.7 in depth (2.3); head length 3.2 in SL (3.1); snout length 3.4 in HL (3.6); bony interorbital width 1.6 in snout length (1.3); orbit diameter 2.8 in HL; post-orbital head length 4.8 in SL (4.7); least depth of caudal-peduncle 2.5 in HL; caudal peduncle length 3.6 in HL (4.0).

Scales ctenoid; lateral line complete and broadly arched over pectoral fin following body contour; 30 (27) tubed scales; scales above lateral line to origin of dorsal fin 2; scales above lateral line to base of middle dorsal spine 2; scales below lateral line to origin of anal fin 9 (10); diagonal rows of scales on cheek 5; scales on top of head extending anteriorly to vertical from anterior margin of pupil; no scales on chin, maxilla, or snout; circumpeduncular scales 10 (11); gill rakers 5+13 (6+13); vertebrae 10+16; supraneurals 3; anterior supraneural-dorsal ray-pterygiophore-neural spine interdigitation pattern: 0/0+0/2/1+1/1.

Mouth large and terminal, slightly upturned; lower jaw protrudes slightly; maxilla expanded posteriorly, extending to below the posterior edge of eye; dorsal profile of head almost straight; upper jaw with one fixed, stout canine on either side of symphysis; upper canines flanked internally by villiform band with four or five rows of depressible, smaller, sharp-tipped teeth; inner rows become progressively longer, innermost row with largest teeth; lower jaw has pair of fixed, short stout canines on either side of symphysis followed by smaller, depressible, sharp-tipped conical teeth in a villiform band of 3–5 rows; lower teeth become progressively longer on inner rows; vomer roughly V-shaped band of two rows of similarly sized, sharp-tipped, conical teeth; palatines with one row of small, sharp-tipped conical teeth; tongue small, slender, pointed, and without teeth.

Opercle with three spines, the middle spine the longest; preopercle with 14 (17) small spines (serrae) along posterior margin; antrorse spines lacking on ventral margin; interopercle with no spines; subopercle smooth, with no spines; anterior nostrils positioned halfway between snout and eye, each with a small rounded flap rising from anterior rim; posterior nostrils an elliptical opening at anterior border of orbit.

#### Color in life.

***Body***: overall white with two rows of bright orange rhomboid-shaped polygons, four to six in each row, arranged in an irregular grid along lateral midline of body; uppermost row of orange polygons proceeds from behind eye to dorsal third of caudal peduncle; lower row starts just dorsal to origin of pectoral fin and continues to ventral half of caudal peduncle; throat and belly white. ***Head***: dorsal third of head orange and bottom two thirds pinkish white with two yellow stripes, both originating from the tip of the upper lip. The first extending horizontally across orbit, bifurcating past posterior edge of pupil to approximately edge of opercle. The second from tip of upper lip, tracing obliquely along maxilla and extending to ventral edge of preopercle. Preopercle region with a yellow triangular patch, from lower mid-orbit expanding in width to edge of preopercle with bifurcations to horizontal edge of operculum and pelvic fin bases respectively; orbit mostly orange-red; iris outlined in silver-grey to black with horizontal yellow stripe through middle of anterior portion; posterior portion of iris with two yellow stripes arising from a bifurcation of the anterior yellow stripe; pupil black. ***Fins***: first three membranes of spinous portion of dorsal fin mostly orange with yellow highlights; third spine with yellow and white membrane; remaining membranes of spinous portion mostly yellow, with hyaline tips; yellow coloration continues on lower third of soft portion of dorsal fin, with upper two-thirds mostly hyaline; some yellow and pale orange on tips of soft dorsal and membranes of last four to five rays; caudal fin hyaline with white and orange rays; anal fin mostly yellow, with white margin; pelvic fins mostly white distally, with yellow rays proximally; pectoral fins hyaline. Living specimen photographed in the Marshall Islands exhibits more yellow coloration on head, within rhomboid-shaped polygons on lateral midline, and on first three membranes of spinous dorsal fin (Fig. 2A).

#### Color in alcohol.

Light tan overall, with no visible markings.

#### Etymology.

*Plectranthias polygonius* sp. nov. is named for the orange rhomboid-shaped polygons arranged in parallel rows along the lateral midline that distinguish it from all other known species within the genus. To be treated as a noun in apposition.

#### Distribution and habitat.

**Figure 3.**
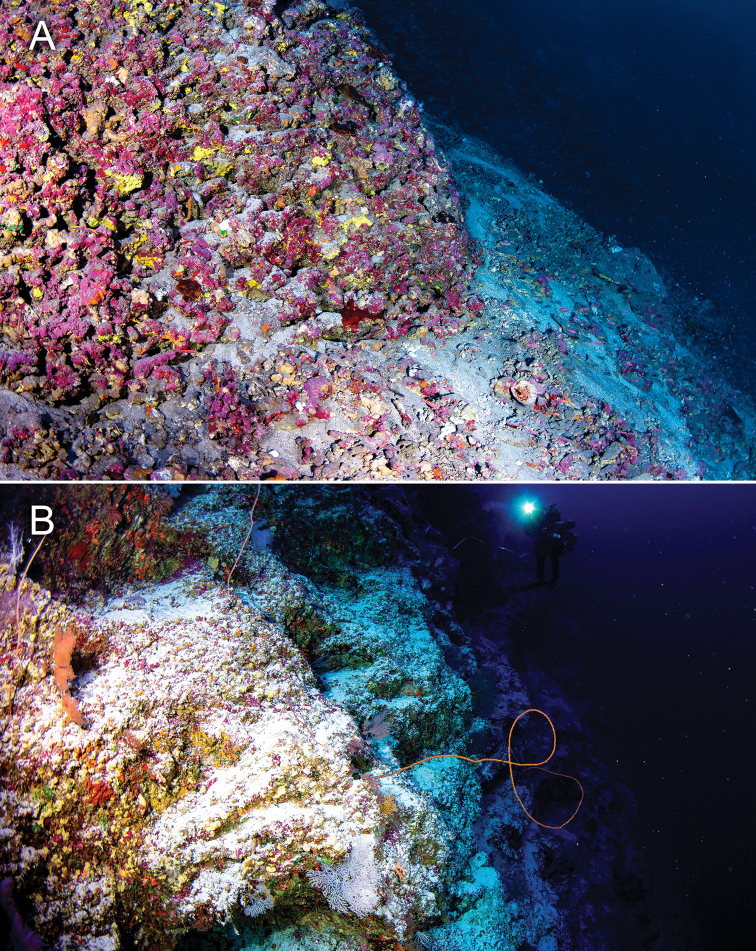
Habitat of *Plectranthias polygonius* sp. nov. and *Plectranthias hinano* sp. nov. in **A** Tahiti, French Polynesia, depth of approximately 100 m, and **B** Erikub Atoll, Republic of the Marshall Islands, depth of approximately 120 m. Photographs by LA Rocha.

*Plectranthias polygonius* sp. nov. appears to be the same species as an undescribed *Plectranthias* species that was photographed off Rangiroa, French Polynesia, at a depth of 65 m (Williams et al. 2013). However, some superficial differences exist between our specimen and the one in Williams et al. (2013), including the thickness of the white lines on the upper body and the color of the iris. These may be due to individual variability. The two specimens described in this paper were collected in highly complex reefs predominantly covered by coralline algae and sponges in Tahiti (Fig. 3A) and crevices of steep reef walls in the Marshall Islands (Fig. 3B), indicating that this species probably has a wider Pacific distribution. All known individuals have been observed or collected at mesophotic depths, suggesting that *Plectranthias polygonius* sp. nov., as with most of its congeners, does not inhabit shallow coral reef habitats.

#### Comparisons.

The general body shape, color, and prolongation of the third dorsal-fin spine in *Plectranthias polygonius* sp. nov. resemble *P. inermis* and *P. altipinnatus* (Fig. 2B, C); however, the barcode fragment of the COI gene of *P. polygonius* sp. nov. is not close to any published barcode sequence of *Plectranthias*, with approximately 15% uncorrected pairwise genetic distance from several species in the genus. Morphologically, it can easily be distinguished from *P. inermis* by having ten circumpeduncular scales (vs. 14 or 15 in *P. inermis*); canine teeth on the lower jaw (lacking in *P. inermis*); and 14–17 spines on the posterior edge of the preopercle (spines lacking in *P. inermis*; feebly serrated in *P. altipinnatus*). The new species differs from *P. altipinnatus* by having X, 16 dorsal-fin rays (X, 18 in *P. altipinnatus*), a shallower body (3.2–3.4 in SL vs. 2.8 in *P. altipinnatus*), smaller head (3.1–3.2 in SL vs. 2.2 in *P. altipinnatus*), and a larger eye (2.8 in HL vs. 4.75 in *P. altipinnatus*).

### Plectranthias hinano sp. nov.

Taxon classificationAnimaliaPerciformesSerranidae

ACE3522C-C99C-5170-B48C-BDE5B44115ED

http://zoobank.org/84A5C4AA-577B-4D79-BE21-8973EFDF7BE9

**Figure 4.**
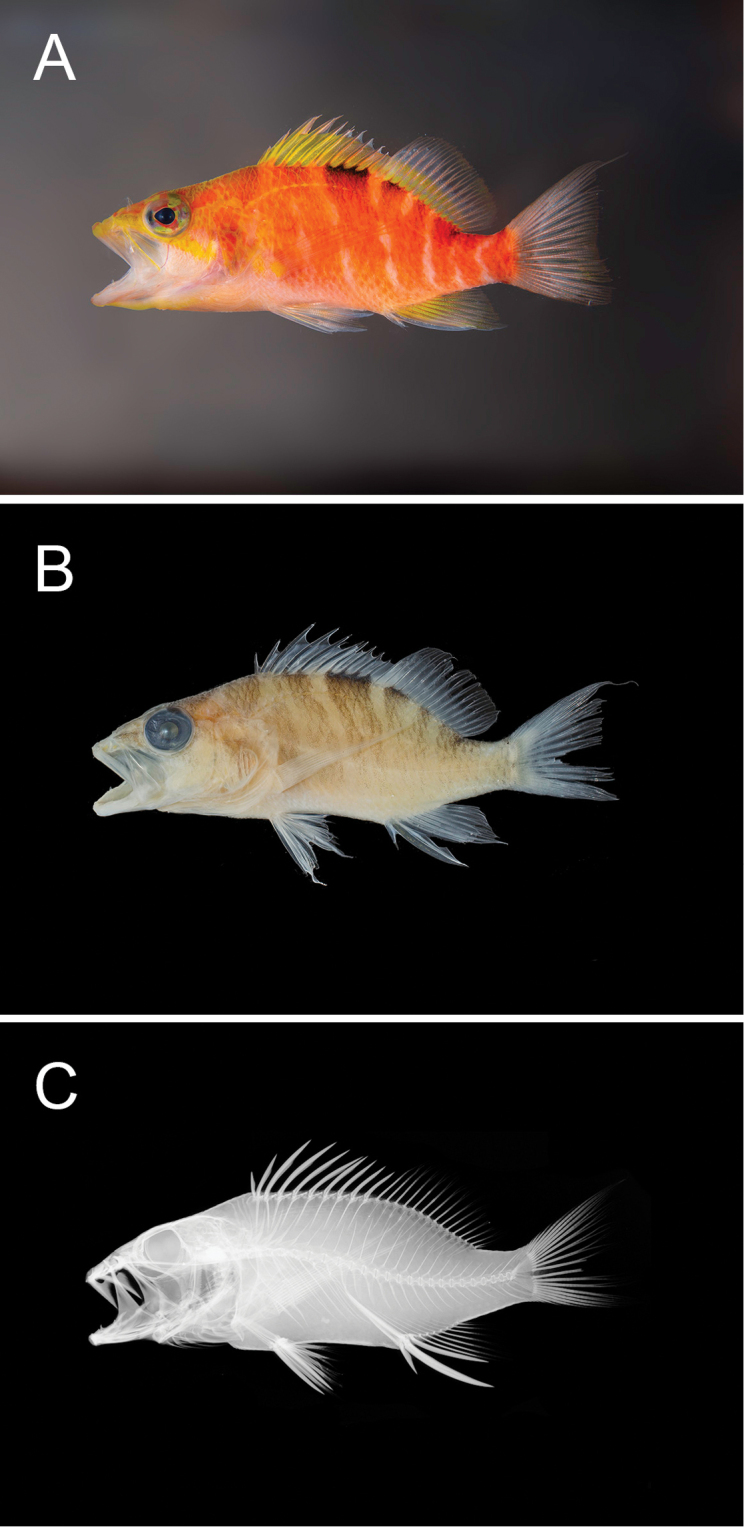
*Plectranthias hinano* sp. nov. holotype **A** shortly after death **B** preserved specimen **C** radiograph. Photographs by LA Rocha (**A**), A Gaisiner (**B**), and J Fong (**C**).

**Figure 5.**
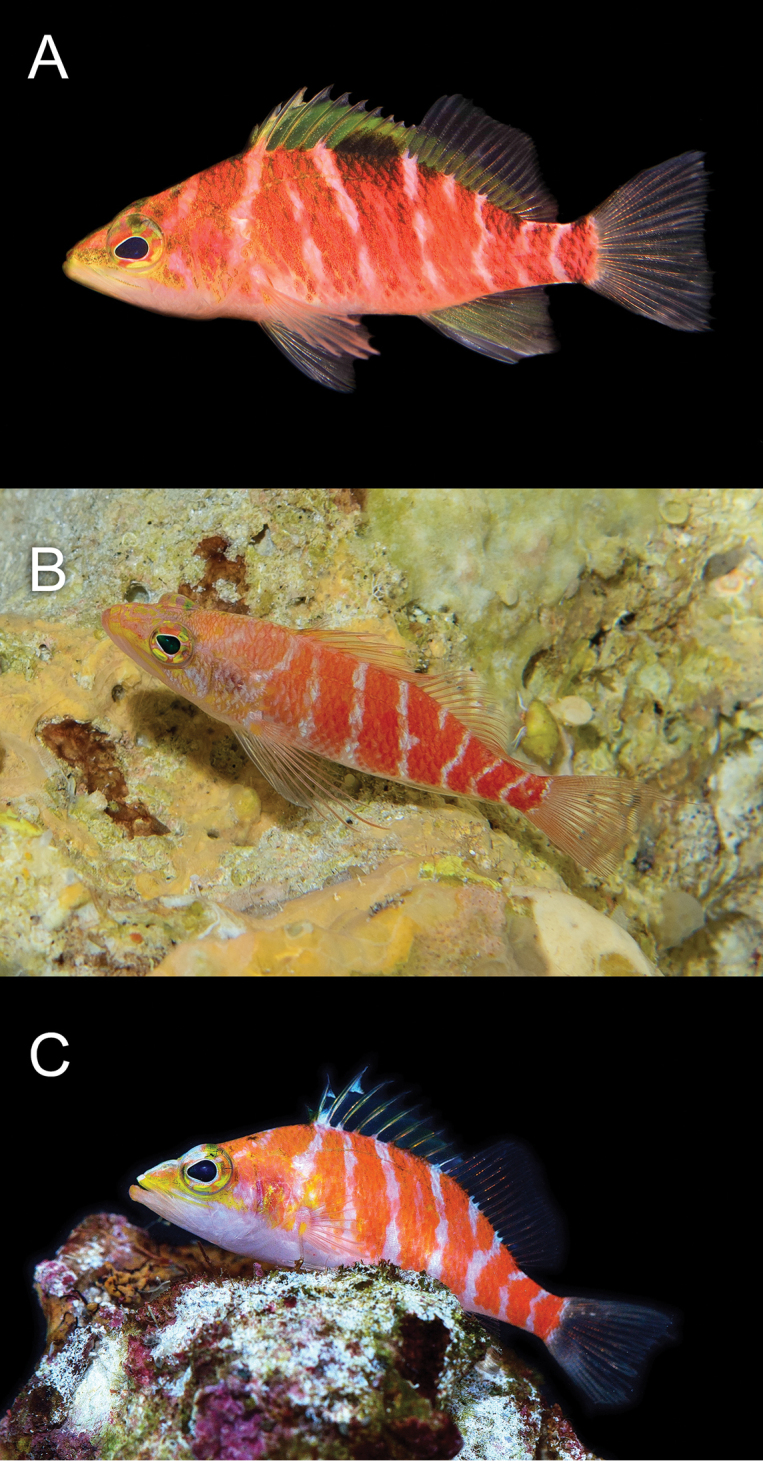
Living specimens of **A***Plectranthias hinano* sp. nov. at Steinhart Aquarium, California Academy of Sciences **B**Plectranthias cf. bennetti photographed at a depth of 120 m at Maloelap Atoll, Republic of the Marshall Islands, and **C***Plectranthias bennetti* photographed in an aquarium. Photographs by T Wong (**A**), LA Rocha (**B**), and YK Tea (**C**).

Figures 4 , 5 , Table 1 Hinano’s Perchlet

#### Type locality.

Tahiti, French Polynesia

#### Holotype.

CAS 247195, field code: HTP909, GenBank MN922329. 49.6 mm SL, Tahiti, French Polynesia, 17°29'27"S, 149°28'01"W, depth of collection 98 m, collected with hand nets by B Shepherd, HT Pinheiro, TAY Phelps, MV Bell, and LA Rocha, 03 March 2019 (Fig. 4).

#### Paratype.

USNM 445723, field code: TAH007, GenBank MN922328. 28.0 mm SL, Tahiti, French Polynesia, 17°36'59"S, 149°37'13"W, depth of collection 90 m, collected with hand nets by B Shepherd, HT Pinheiro, TAY Phelps, MV Bell, and LA Rocha, 28 February 2019.

#### Diagnosis.

*Plectranthias hinano* sp. nov. can be distinguished from all of its congeners by the following combination of characters: dorsal-fin rays X, 15, the last branched to base and counted as one; pectoral fin rays 11 or 12; vertebrae 10+16; lateral line complete with 29–30 tubed scales; gill rakers 7–8+12–13; ventral margin of preopercle with three antrorse spines; snout moderately long, 3.0–3.2 in HL, 12.3–14.2% SL, overall red coloration with two indistinct black spots along the base of dorsal fin, translucent yellow dorsal and anal-fin membranes.

#### Description.

Proportional measurements for the type specimens are presented in Table 1. Dorsal-fin rays X, 15; last soft ray branched to base and counted as one; first dorsal spine very short, 17.6 in SL (9.4); fourth dorsal spine longest, 5.5 in SL (third dorsal spine longest, 6.4 in SL); dorsal-fin base length 2.1 in SL (1.9); anal-fin rays III, 7; last soft ray branched to base and counted as one; anal-fin base 6.2 in SL (5.3); second anal spine longest and stoutest at 2.0 in HL (2.1); anal-fin origin at vertical beneath fourth dorsal-fin ray; pectoral-fin rays 11 (12), all unbranched, length 2.8 in SL (2.6); pelvic fin I, 5; pelvic-fin length 4.6 in SL (4.0); pelvic-spine length 2.4 in HL (2.3); caudal-fin procurrent rays 6+5; caudal-fin principal rays 9+8.

Body moderately elongate, laterally compressed; depth of body 2.9 in SL (3.0); width of body 2.1 in depth (2.4); head length 2.4 in SL (2.5); snout length 3.0 in HL (3.2); bony interorbital width 3.5 in snout length (2.3); orbit diameter 3.7 in HL (3.5); post-orbital head length 5.4 in SL (5.6); least depth of caudal-peduncle 3.7 in HL (3.4); caudal-peduncle length 4.0 in SL (4.1).

Scales ctenoid; lateral line complete and broadly arched over pectoral fin following body contour; 29 (30) tubed scales; scales above lateral line to origin of dorsal fin 3; scales above lateral line to base of middle dorsal spine 2; scales below lateral line to origin of anal fin 12; diagonal rows of scales on cheek 4; scales on top of head extending anteriorly to vertical from center of eye; area on top of head between eyes with scales; no scales on chin, maxilla, or snout; circumpeduncular scales 12; gill rakers 8+13 (7+12); vertebrae 10+16; supraneurals 3; anterior supraneural-dorsal ray-pterygiophore-neural spine interdigitation pattern: 0/0+0/2/1+1/1.

Mouth large and terminal, slightly upturned; lower jaw protrudes slightly; maxilla expanded posteriorly, extending to below the posterior edge of pupil; upper jaw with one fixed, stout canine on either side of symphysis; upper canines flanked internally by villiform band with 7–9 irregular rows of depressible, smaller, sharp-tipped teeth; inner rows become progressively longer, innermost row with largest teeth; lower jaw has one large, fixed canine on either side of lower jaw, roughly at midpoint, followed by smaller, depressible, sharp-tipped conical teeth in a villiform band of 4–6 irregular rows, innermost teeth same size as outer rows; vomer roughly V-shaped band of two rows of similarly-sized, sharp-tipped, conical teeth; palatines with one row of small, sharp-tipped conical teeth anteriorly, two rows posteriorly; tongue small, slender, pointed, and without teeth.

Opercle with three flat spines, the middle spine the longest; preopercle with 22 (12) small spines (serrae) along posterior margin; ventral (inferior) margin of preopercle with three antrorse spines; interopercle with no spines; subopercle smooth, with one spine on inferior margin; anterior nostrils positioned closer to eye than to snout, each with a small rounded flap rising from anterior rim; posterior nostrils an elliptical opening at anterior border of orbit.

#### Color in life.

***Body***: overall light red in color; chest and belly mostly light red to pink; dorsal portion of body darker red with yellow along scale margins and lateral line; series of 8–12 pink to white incomplete bars on the body, originating just behind orbit and continuing to base of caudal fin; bars are approximately 20 degrees off of vertical anteriorly, becoming near vertical as they approach base of caudal fin; black spot, almost twice the diameter of orbit, at base of spinous dorsal-fin spines 7–10 and soft rays 1–5, continuing slightly more than halfway up membrane of spinous dorsal; black spot is interrupted by the near-vertical white bar originating below soft rays 1 or 2; smaller second black spot, slightly smaller than orbit, located at base of soft dorsal rays 11–15. ***Head***: snout, throat, anterior portion of lower lip, maxilla, and operculum mostly light pink; yellow stripe originating at snout, proceeding across maxilla, below orbit to preopercle; lattice-like network of indistinct yellow stripes radiating outward from pupil across iris, between eyes, across top of head, from ventral margin of orbit to origin of lateral line and lower margin of operculum; iris mostly pink with yellow splotches radiating outward from pupil; pupil black and teardrop-shaped, pointed anteriorly. ***Fins***: spinous portion of dorsal fin predominantly translucent yellow, with upper half of black spot on membrane between spines 7–10; lower third of soft dorsal fin mostly translucent yellow, upper two-thirds hyaline; caudal-fin membranes mostly hyaline with some regions a faint transparent yellow, fin rays white with red margins; pelvic fins hyaline with faint yellow on rays; anal fin mostly translucent yellow with hyaline margins; pectoral fins hyaline with rays outlined in pink; base of pectoral fins yellow. Freshly dead specimens exhibit similar coloration, with slightly more yellow on head, body, and fins (Fig. 4A).

#### Color in alcohol.

Light tan overall, with dark brown stippling in vertical bands along lateral sides of body, darkest brown along base of dorsal fin at location of black spots.

#### Etymology.

*Plectranthias hinano* sp. nov. is named after Teurumereariki Hinano Teavai Murphy, former associate director of the University of California Berkeley Gump Research Station and president of the cultural association Te Pu Atitia, in honor of the significant contributions she has made supporting Polynesian biocultural heritage and field research in Moorea, French Polynesia. The name is a noun in the genitive case.

#### Distribution and habitat.

The two specimens described in this paper, plus an individual that was retained for public aquarium exhibition, were collected respectively in Tahiti and Moorea, French Polynesia. A similar species was photographed at 120 m depth in Erikub Atoll, Republic of Marshall Islands, however specimens were not retained (Fig. 5B). The Marshall Islands specimens lack the black markings along the dorsal-fin base, and thus more closely resemble *Plectranthias bennetti* (Fig. 5C), indicating that the latter may have a wider Pacific distribution and not just be restricted to the Coral Sea. All known individuals of *P. hinano* sp. nov. have been observed or collected in highly complex habitats on walls and ledges within MCEs (Fig. 3), suggesting that the species does not inhabit shallow coral reefs.

#### Comparisons.

The most similar barcode fragment of the mtDNA COI gene to *Plectranthias hinano* sp. nov. is *P. bennetti* from the Coral Sea (5.5% uncorrected pairwise genetic distance). Morphologically, it can be distinguished from *P. bennetti* by having a longer snout (3.0–3.2 in HL vs. 4.4 in *P. bennetti*), a larger orbit (3.5–3.7 in HL vs. 4.1 in *P. bennetti*), in the number of circumpeduncular scales (12 vs. 14 in *P. bennetti*), the number of gill rakers (7–8 + 12–13, vs. 5+13 in *P. bennetti*), and in coloration (by having two indistinct black spots along the base of the dorsal fin, and yellow dorsal and anal fin membranes).

## Discussion

Species within the genus *Plectranthias* appear to be common and conspicuous inhabitants of MCEs, and we have regularly observed them in highly complex, rocky habitats with abundant coralline algae, sponges, and black corals (Fig. 3). They are usually sampled with the use of hand nets and closed-circuit rebreather technical diving. As with much of the Anthiadinae, the genus *Plectranthias* is in need of revision, as it is not currently defined on the basis of synapomorphies, and there is high variation within many of the defining characters (Anderson and Heemstra 2012; Gill et al. 2016). Hence, placement of the two new species presented here should be regarded as provisional. We expect that the known diversity within *Plectranthias* will continue to expand with further MCE exploration, and that future sampling, comparative work, and genetic analysis will unravel some of the taxonomic confusion within this genus.

More than 70% of all research on MCEs has been published in the past seven years (Pyle and Copus 2019), and undoubtedly these discoveries will continue as several teams lead expeditions to global biodiversity hotspots and regions where MCEs have not been previously surveyed. Continued research on mesophotic coral ecosystems (MCEs), whether using submarines, closed-circuit rebreathers or ROVs, is documenting many new species and range extensions for fishes found at depths between 60–150 m (Pyle 2000; Baldwin et al. 2016; Pinheiro et al. 2016, 2019; Pyle et al. 2016; Rocha et al. 2017; Shepherd et al. 2018; Arango et al. 2019; Shepherd et al. 2019). Discovery rates of new species are as high as two new species per hour of exploration (Pinheiro et al. 2019). This, coupled with ecological observations, has led to the conclusion that mesophotic coral reefs are unique and threatened (Baldwin et al. 2018; Rocha et al. 2018). Through research expeditions using technical diving and closed-circuit rebreathers to systematically survey habitats down to 150 m depth across tropical regions of the world, the California Academy of Sciences’ Hope for Reefs initiative is advancing the knowledge of the biodiversity and ecology of MCEs. Our goals are not only to document undiscovered species diversity, but also to raise awareness of the need to protect MCEs from anthropogenic impacts such as climate change, unsustainable fishing practices, plastic pollution, and sedimentation.

### Comparative material

*Plectranthias inermis*. CAS 241326. Philippines, Batangas, 28 April 2015.

*P. japonicus*. CAS 33555, 1. Philippines, Batangas, 23 June 1966.

*P. longimanus*. CAS 213185, 3. Fiji, Viti Levu Island, 31 May 1999.

*P. sagamiensis*. CAS 235596, 1. Philippines, Luzon Island, 02 June 2011.

*P. winniensis*. CAS 219169, 1. Fiji, Vanua Levu Island, 27 May 2003.

## Supplementary Material

XML Treatment for Plectranthias polygonius

XML Treatment for Plectranthias hinano
